# Improved motor imagery training for subject’s self-modulation in EEG-based brain-computer interface

**DOI:** 10.3389/fnhum.2024.1447662

**Published:** 2024-08-26

**Authors:** Yilu Xu, Lilin Jie, Wenjuan Jian, Wenlong Yi, Hua Yin, Yingqiong Peng

**Affiliations:** ^1^School of Software, Jiangxi Agricultural University, Nanchang, China; ^2^School of Measuring and Optical Engineering, Nanchang Hangkong University, Nanchang, China; ^3^School of Information Engineering, Nanchang University, Nanchang, China

**Keywords:** motor imagery training, brain-computer interface, trial-feedback paradigm, run evaluation, self-modulation

## Abstract

For the electroencephalogram- (EEG-) based motor imagery (MI) brain-computer interface (BCI) system, more attention has been paid to the advanced machine learning algorithms rather than the effective MI training protocols over past two decades. However, it is crucial to assist the subjects in modulating their active brains to fulfill the endogenous MI tasks during the calibration process, which will facilitate signal processing using various machine learning algorithms. Therefore, we propose a trial-feedback paradigm to improve MI training and introduce a non-feedback paradigm for comparison. Each paradigm corresponds to one session. Two paradigms are applied to the calibration runs of corresponding sessions. And their effectiveness is verified in the subsequent testing runs of respective sessions. Different from the non-feedback paradigm, the trial-feedback paradigm presents a topographic map and its qualitative evaluation in real time after each MI training trial, so the subjects can timely realize whether the current trial successfully induces the event-related desynchronization/event-related synchronization (ERD/ERS) phenomenon, and then they can adjust their brain rhythm in the next MI trial. Moreover, after each calibration run of the trial-feedback session, a feature distribution is visualized and quantified to show the subjects’ abilities to distinguish different MI tasks and promote their self-modulation in the next calibration run. Additionally, if the subjects feel distracted during the training processes of the non-feedback and trial-feedback sessions, they can execute the blinking movement which will be captured by the electrooculogram (EOG) signals, and the corresponding MI training trial will be abandoned. Ten healthy participants sequentially performed the non-feedback and trial-feedback sessions on the different days. The experiment results showed that the trial-feedback session had better spatial filter visualization, more beneficiaries, higher average off-line and on-line classification accuracies than the non-feedback session, suggesting the trial-feedback paradigm’s usefulness in subject’s self-modulation and good ability to perform MI tasks.

## 1 Introduction

A brain-computer interface (BCI) system can directly build a two-way communication path between a subject’s brain and an electrical device without the participation of motor muscles (Wolpaw et al., 2002). Among various BCI paradigms, motor imagery (MI) has been paid great attention due to its ability to improve motor functions for patients with motor nerve disorder (Mane et al., 2020). Moreover, because of high temporal resolution and reliable safety, electroencephalography (EEG)-based MI-BCI has been widely studied in real-time applications.

Nevertheless, EEG signals are of non-stationarity, low spatial resolution, and poor signal-to-noise ratio (SNR). Furthermore, it is tough for patients and even healthy subjects to execute MI tasks since they can naturally control their limbs with their peripheral nerves but hardly know how to imagine the movements of their limbs. Statistically, about 30% of subjects fail to reach a benchmark classification accuracy (*CA*) of 70% in real-time BCI control. This phenomenon is coined as BCI illiteracy (Vidaurre and Blankertz, 2010). Moreover, MI-BCI is an asynchronous system, which spontaneously initiates a manipulation at any time without any external stimuli. Therefore, a long MI training is needed to collect amounts of steady and reliable brain signals for a subject-specific classifier, which unfortunately might cause the subject’s fatigue and the experimental results’ degeneration. Consequently, it is a challenge to shorten the MI calibration time without sacrificing the performance of EEG-based MI-BCI.

Many advanced machine learning algorithms have been ventured into investigating how to find a way out of this dilemma, such as semi-supervised learning (Ko et al., 2020; Zhang et al., 2023), transfer learning (Wan et al., 2021; Wu et al., 2022), and semi-supervised transfer learning (Zhao et al., 2019; Gao and Sun, 2022). Despite sophisticated machine learning algorithms, their performance partially relies on the quality of available samples. An efficient training procedure is helpful to generate a labeled set with good between-class discrepancy. To tackle this problem, Jeunet et al. (2016) called for updating the standard BCI training protocol, which has been widely used in MI-BCI over a long period of time. In traditional Graz training protocol, subjects repeatedly and passively perform left-hand and right-hand MI tasks instructed by the auditory and visual cues to collect amounts of labeled data (Pfurtscheller et al., 1993). The whole calibration process is short of involvement, feedback, and adjustment, resulting in a bad experience sense for the subjects.

To solve this problem, there exists many variants of standard training protocol. To increase the subject’s interest, the dull arrow cues on the computer screen were replaced by the vivid visual cues, such as a yellow spaceship navigating through a galaxy (Abu-Rmileh et al., 2019), a moving hand which can write Chinese characters (Qiu et al., 2017). Questionnaire can not only obtain the subject’s feedback on the experiment but also foster the subject’s involvement, whose role has been always underestimated. Ahn et al. (2018) pointed out that the subject’s self-predicted BCI performance in pre-task questionnaire showed a high positive correlation with the actual accuracy, suggesting that the subject’s initiative can influence the experimental results. However, questionnaire cannot provide further feedback about subject’s MI-BCI skill. *CA*, the commonly used metric of BCI performance, is traditionally applied to the testing runs. Thus, *CA* is not the most suitable metric to quantify MI-BCI skill during the calibration phase. Lotte and Jeunet (2018) defined the binary class distance/stability of EEG samples to assess the progress or retrogression of subject’s MI-BCI skill in different calibration runs. Nevertheless, such subjective feedback (questionnaire) and objective feedback (*CA* and class distance/stability) cannot provide real-time information to help the subjects realize self-modulation in the training phase.

Based on event-related desynchronization (ERD)/event-related synchronization (ERS) phenomenon (Pfurtscheller et al., 2000), Hwang et al. (2009) presented a real-time brain activation map during the calibration process. However, the subjects might be confused by the complex topographic map since it was difficult for them to correctly activate all electrodes. Bai et al. (2014) provided a more concise perspective in which the cortical ERD features of four electrodes (C1, C2, C3, and C4) were presented in the form of four column panels. Each panel included a blue bar representing the real-time EEG activity and a red bar denoting the baseline EEG amplitude. Nevertheless, this training protocol lost the ERD characteristics of other electrodes over the sensorimotor area. Due to the importance of class discrepancy, Duan et al. (2021) exhibited an online data visualization where the EEG distribution in Riemannian geometry appeared on a computer monitor. However, it is challenging to remain the valuable information of high-dimensional EEG data on a two-dimensional screen. Vasilyev et al. (2021) modified the training protocol in which the subjects were presented with the classification result of current EEG trial in real time. Mladenović et al. (2022) provided three MI training protocols with different feedback for comparison: no bias feedback, positive bias feedback, and negative bias feedback, in which the classifier result was not biased, positively and negatively biased in real time, respectively. Nevertheless, these training protocols overly relies on the classifier output.

Recently, many external devices have presented various feedback to improve the subject’s self-modulation in the MI-BCI system. Proprioceptive feedback provided by the two orthoses can facilitate MI-related operant learning (Darvishi et al., 2017). Vibration stimulation accompanied with the traditional visual cue of the MI task promoted the activation of sensorimotor rhythm (Zhang et al., 2022). Tactile feedback was given by a force sensor in an MI-based robot control system or a neurofeedback training system (Xu et al., 2020; Marcos-Martínez et al., 2021). Immersive feedback can increase feedback visualization through the virtual reality device (Škola et al., 2019; Choi et al., 2020; Achanccaray et al., 2021). Multisensory (such as audio, visual, tactile) feedback can further boost the training effect (Wang et al., 2019; Li et al., 2022). Nevertheless, their corresponding methods generally divided the whole MI training process into two phases. The former phase was used to train the initial classifier without feedback, while the latter phase was used to assist the subjects to adjust themselves with such embodied feedback. Therefore, it is still difficult for these methods to enhance the quality of the initial training samples.

Inspired by such studies, we aimed to provide prompt feedback to help the subjects timely realize whether their EEG signals conformed to the ERD/ERS feature during the whole calibration phase. Thus, we proposed a trial-feedback paradigm in which a topographic map and an objective evaluation were simultaneously given after each MI training trial to foster the subject’s self-modulation. Furthermore, we presented a run evaluation after each calibration run to assess the subjects’ abilities to distinguish different classes. To investigate the effect of our trial-feedback paradigm, we designed the non-feedback session and the trial-feedback session for comparison. Both sessions included the electrooculogram (EOG), calibration, and testing runs. The goal of EOG run was to collect the signals from electrode Fp2 triggered by the blinking movements. In the subsequent calibration runs, the EOG signal from the subject can be translated into a command of accepting or refusing the current training trial, leading to the improvement of the subject’s involvement. The differences between the calibration runs of two sessions lied in whether there were prompt feedback and run evaluation. The testing runs were used to testify the training samples’ quality and the classifier’s performance. Moreover, concise questionnaires were given to record the subject’s pre- and post-experimental status.

Our contributions can be outlined as follows. First, we visualized and evaluated the subject’s MI-BCI skills using prompt feedback and run evaluation. Secondly, we utilized the EOG signals to enhance the subject’s involvement in the MI training process. Thirdly, we arranged a complete MI experiment including the calibration and testing runs to testify the effectiveness of MI training. Finally, we summarized the pros and cons of our trial-feedback paradigm and identified the future areas of improvement in MI-BCI experimental design.

## 2 Methods

### 2.1 Subjects

Ten healthy subjects (S1–S10, 1 female and 9 males, aged 22–43 years, mean 24 ± 6.8) participated our MI experiment. Five subjects (S6–S10) had no MI experience before this study. All subjects signed an informed consent in accordance with the Declaration of Helsinki before participating the experiment. The experimental procedure was approved by the ethics committee of Jiangxi Agricultural University. All subjects were right-handed with normal neurological examinations and finished the whole experiment in a quiet room.

### 2.2 EEG acquisition

**Figure 1:**
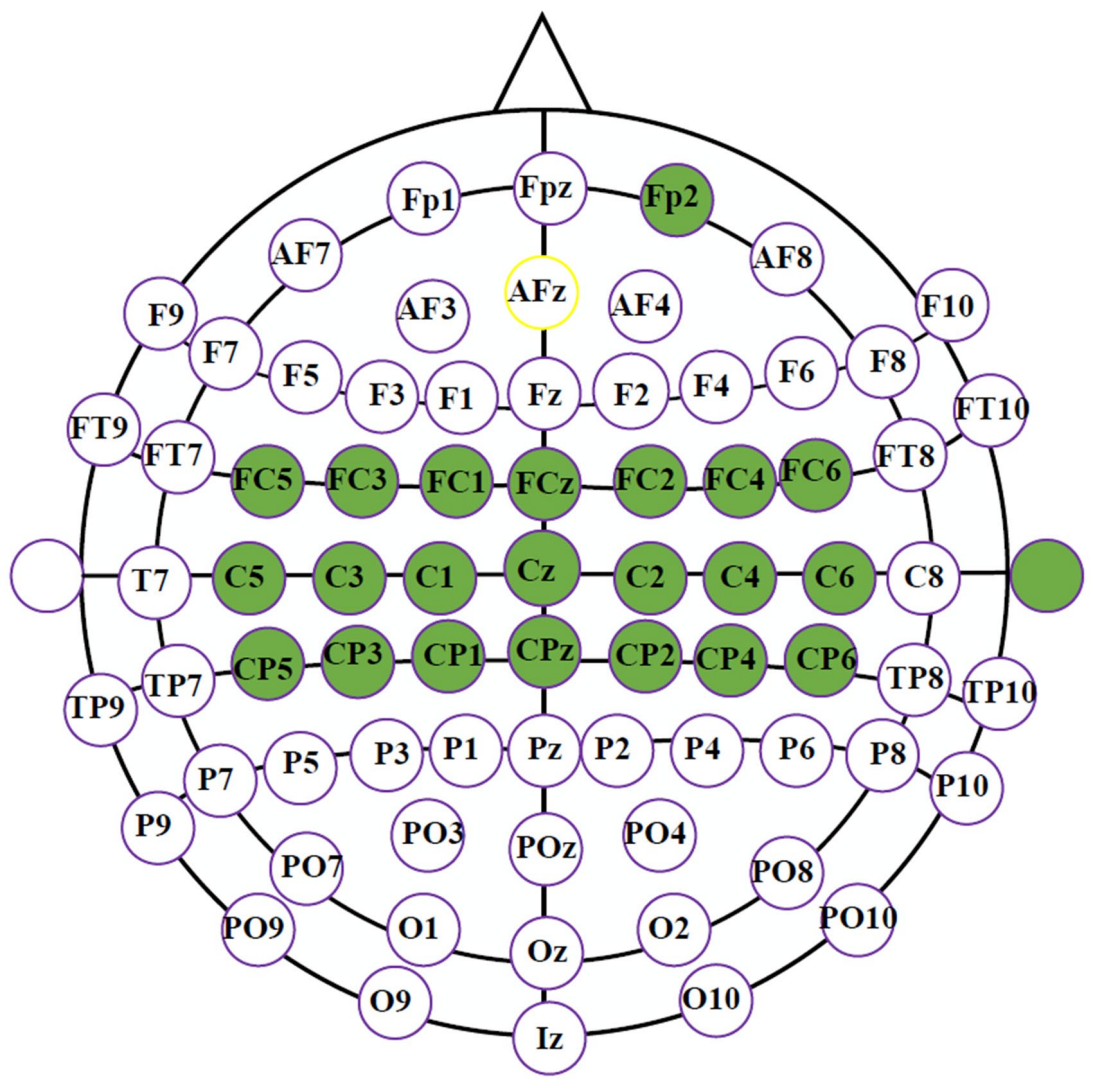
The selected electrodes in our MI experiment.

EEG signals were collected with a g. Hlamp amplifier at 256 sampling rate. The neural activities were recorded through 21 electrodes over the sensorimotor cortex using a 64-electrode cap following the 10–20 international system. EOG signals were acquired by Fp2 at the prefrontal cortex to improve the subject’s involvement. In Figure 1, all selected electrodes are marked in green, referenced to the right ear and grounded to AFz.

### 2.3 Experimental design

**Figure 2:**
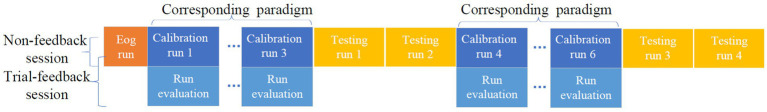
The experimental design on different sessions.

To explore the role of feedback, the non-feedback and trial-feedback sessions were developed for comparison and were sequentially executed on different days for each subject. Each session consists of one EOG run, six calibration runs, and four testing runs. The EOG run was performed before the calibration runs to acquire the EOG features of subject’s blinking movements. The calibration runs of different sessions were conducted to collect training data using corresponding paradigms. During the calibration run, if the subject felt distracted, he/she could mark the current trial as dissatisfied one by blinking. Additionally, a run evaluation was performed after each calibration run of the trial-feedback session. To evaluate the effect of training, a simple brain-controlled Belly-Worship snake game, with a snake moving right or left, was played in the subsequent testing runs. The experimental design on different sessions is shown in Figure 2.

As illustrated in Figure 2, we additionally performed a run evaluation which aimed to visualize and quantify the subject’s competency after each calibration run of trial-feedback session. Moreover, the trial-feedback session provided prompt feedback after each MI training trial. To investigate the impact of the size of training set on the model, the first two and last two testing runs were executed after the three and six calibration runs, respectively. More details can be seen in the following sections.

### 2.4 EOG run

**Figure 3:**
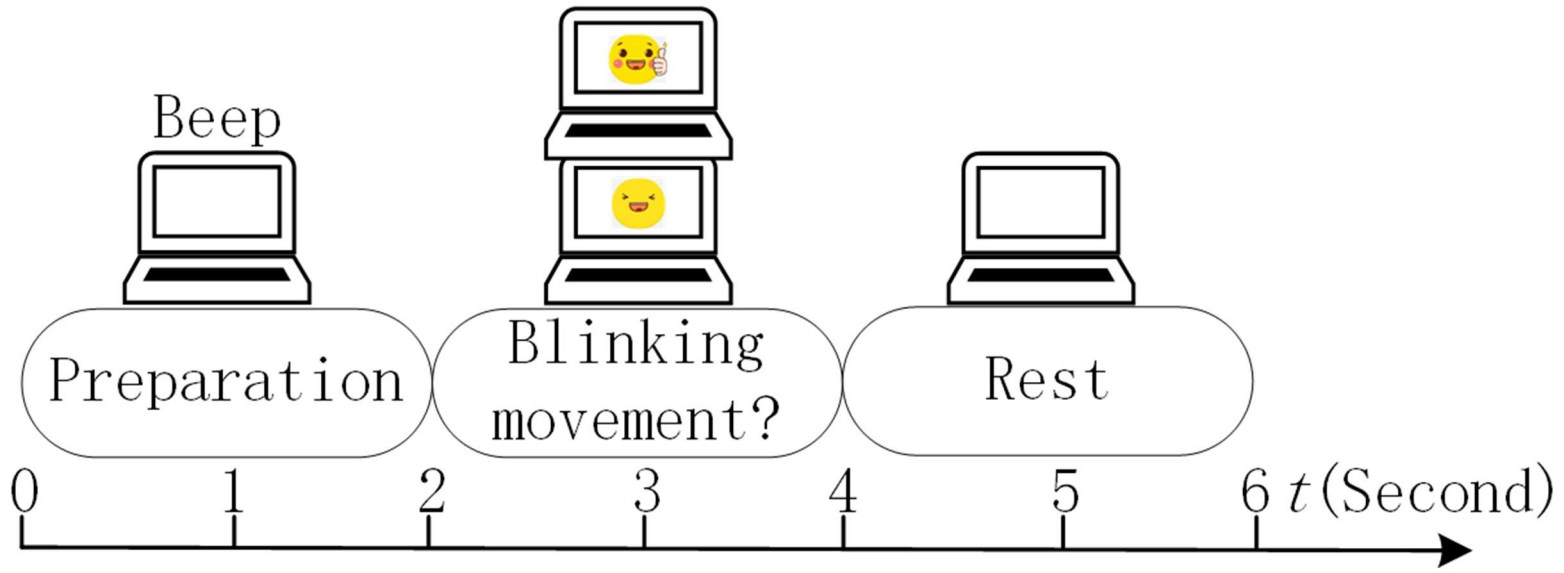
Timing scheme of a trial in the EOG run.

In our MI experiment, EOG signals were utilized to enhance the subject’s involvement because of their high SNR. In the traditional MI paradigm, the subject cannot do anything if he/she feels distracted, which may lead to the degeneration of the model. Inspired by active learning (Tharwat and Schenck, 2023), our subjects could mark the distracted trials unsatisfied by blinking during the calibration run. Thus, the EOG run was used to collect EOG features of blinking and unblinking movements. Each EOG run consisted of 20 trials of 6 s each. The timing scheme of a trial in the EOG run is shown in Figure 3.

The timing scheme included the preparation, blinking movement, and rest phases. After 2 s of preparation and a short beep, a picture with two eyes opening or blinking randomly appeared on the screen for 2 s, directing the subject to execute the desired eye task.

Because of stronger eye movement, the blinking movement usually has a higher amplitude than the unblinking one. The peak of 2-s data epoch was thus regarded as the feature of blinking trial. The outliers of blinking trials were detected depending on the median value of the blinking features. The blinking trials with higher (>2 × median) or lower (<median/2) features were removed. The mean of remaining blinking features was used as the threshold of blinking movements in the calibration run.

### 2.5 Calibration run

#### 2.5.1 Time scheme

**Figure 4:**
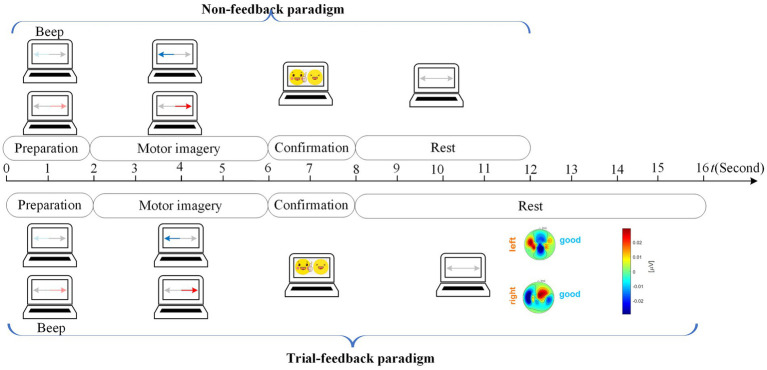
Timing scheme of a trial in the calibration run for different paradigms.

In the calibration run, we designed the non-feedback and trial-feedback paradigms for comparison. In Figure 4, the timing scheme of a trial in the calibration run for different paradigms is composed of the preparation, motor imagery, confirmation, and rest phases.

At the beginning of a trial (*t* = 0 s), a baby blue arrow on the left or a light red arrow on the right reminded the subject to prepare the left-hand or right-hand MI task without collecting the neural signals. After 2 s and a short beep, a blue or red arrow appeared on the screen for 4 s while the brain activities evoked by the left-hand or right-hand MI task were recorded from 21 electrodes. During the 2-s confirmation phase, the EOG signal was acquired from Fp2 to detect whether the subject executed blinking movement due to distraction. In the rest phase, the arrows on the two sides became grey.

No feedback was further provided for the non-feedback paradigm whereas a topographic map with a “good” or “fight” hint was presented on the screen to evaluate the current MI training trial for the trial-feedback paradigm. There was adequate time for the subjects to think about how to adjust their MI behavior in the next MI task.

#### 2.5.2 Prompt feedback mechanism

In our experiment, the subject-selected imagery movements of limbs, such as throwing the balls, grasping the mobile phone, cutting the paper, etc., are allowed for all paradigms. As shown in Figure 4, for the trial-feedback paradigm, a topographic map with an objective evaluation appears in the rest phase, which can assist the subjects to understand whether the current imaginative behavior is good or bad, thereby helping them adjust their attention or the amplitudes of their imagination movements.

Such topographic map was presented by modifying a bbci toolbox which can be found at: https://github.com/bbci/bbci_public. This toolbox can present the topographic map per MI class, which shows the average amplitudes of selected electrodes on a given temporal interval. To provide prompt feedback, our topographic map can show the topographic map per MI trial with a slight modification to this toolbox.

To encourage subject, we extracted three temporal segments [(0.5, 1.5), (0.5, 2.5), and (0.5, 3.5)] from the 4-s MI execution time interval, then correspondingly generated three topographic maps, and finally selected the most representative map to show on the screen accompanied with an objective evaluation.

To show the ERD/ERS feature, the *i*-th EEG trial $X_{i}\;\left(X_{i}\in R^{N_{t}\times 21}\right)$ was transformed into ${\dot{X}}_{i}$ $\left({\dot{X}}_{i}\in R^{N_{t}\times 21}\right)$ after executing local average reference, computing the envelop curve of oscillatory signals, and subtracting a baseline, where $N_{t}$ is the number of sample points of $X_{i}$ . Then, the average amplitude over the *k*-th ( $k\in \left[1,2,3\right]$ ) temporal segment at the electrode *j* (*j* ∈J,J= [FC5, FC3, FC1, FCz, FC2, FC4, FC6, C5, C3, C1, Cz, C2, C4, C6, CP5, CP3, CP1, CPz, CP2, CP4, CP6]) was calculated as ${R^{j}}_{k}=\frac{1}{N_{t_{k}}}\sum_{t}{{\dot{X}}_{i}^{t}}^{j}$ , where $N_{t_{1}}=$ (1.5 − 0.5) × 256, $N_{t_{2}}=$ (2.5 − 0.5) × 256, $N_{t_{3}}=$ (3.5−0.5) × 256, and ${{\dot{X}}_{i}^{t}}^{j}$ is the amplitude of the *t*-th sample point of ${\dot{X}}_{i}$ at electrode *j*. Consequently, three topographic maps were obtained using 21 electrodes’ ${R^{j}}_{k}$ .

Next, the representative topographic map was selected depending on the number of correctly activated electrodes. In our experiment, except for FCz, Cz, and CPz, the remaining electrodes on the sensorimotor area were further grouped into left and right sets: *J*_1_ = [FC5, FC3, FC1, C5, C3, C1, CP5, CP3, CP1] and *J*_2_ = [FC2, FC4, FC6, C2, C4, C6, CP2, CP4, CP6]. Based on the characteristics of ERD and ERS, for the left-hand MI trial, the average amplitudes of electrodes on the left cortex should be larger than zero and those on the right cortex should be less than zero. On the contrary, for the right-hand MI trial, the average amplitudes of electrodes on the right area should be positive and those on the left area should be negative. However, due to BCI illiteracy, it is difficult for each electrode to be correctly activated. Therefore, we counted the number of correctly activated electrodes for three topographic maps as below.

##### ALGORITHM 1: The representative topographic map selection

**Input**: the average amplitudes of all electrodes from three topographic maps: ${R^{j}}_{k}$ ( $k\in \left[1,2,3\right]$ and j∈J ).
**Outpu**t: the K -th representative topographic map.
Initialize: *bestMetric* = 0, *flag* = 0;
**
for** k = 1 to 3 **do**
**if** the *i*-th trial belongs to the left-hand MI type **then**
Count the number of correctly activated electrodes on the left cortex: $N_{l}=\mathrm{sum}\left({R^{j}}_{k}>0\right)\left(j\in J_{1}\right)$ ;
Count the number of correctly activated electrodes on the right cortex: $N_{r}=\mathrm{sum}\left({R^{j}}_{k}<0\right)\left(j\in J_{2}\right)$ ;
**
if** $N_{l}>0$ and $N_{r}>0$ **then** *metric* $=N_{l}+N_{r}$ ; *flag* = 1;
**
elseif** $N_{l}>0$ and *flag* == 0 **then** *metric* = $N_{l}$ ;
**
end if
**
**elseif** the *i*-th trial belongs to the right-hand MI type **then**
Count the number of correctly activated electrodes on the left cortex: $N_{l}=\mathrm{sum}\left({R^{j}}_{k}<0\right)\left(j\in J_{1}\right)$ ;
Count the number of correctly activated electrodes on the right cortex: $N_{r}=\mathrm{sum}\left({R^{j}}_{k}>0\right)\left(j\in J_{2}\right)$ ;
**
if** $N_{l}>0$ and $N_{r}>0$ **then** *metric* $=N_{l}+N_{r}$ ; *flag* = 1;
**
elseif** $N_{r}>0$ and *flag* == 0 **then** *metric* = $N_{r}$ ;
**
end if
**
**
end if
**
**
if** *metric* > *bestMetric* **then** *bestMetric* = *metric*; K=k ; **end if**
**
end for
**
**
if** *bestMetric* == 0 **then** select the second topographic map by default: K=2 . **end if**

In Algorithm 1, if the *k*-th map has the correct ERD/ERS phenomenon on the left and right cortices, i.e., $N_{l}>0$ and $N_{r}>0$ , this map will be selected with higher priority. Otherwise, if the *k*-th map only has ERS feature on the left or right cortex, i.e., $N_{l}>0$ or $N_{r}>0$ , this map will be chosen with lower priority. Finally, we chose the best map with maximum number of correctly activated electrodes to show.

After selecting the suitable topographic map based on the number of correctly activated electrodes, an objective evaluation was presented by comparing the selected map’s two topographic values: $\mathrm{TopoVa}l_{1}$ and $\mathrm{TopoVa}l_{2}$ (1 meant right-hand MI type and 2 was left-hand MI type).


(1) evaluation={'good'ifmaxTopoVal1TopoVal2==TopoValcandc∈12'fight'ifmaxTopoVal1TopoVal2≠TopoValcandc∈12,

where


(2) $$\begin{array}{l} {\mathrm{TopoVal}}_{1}=\\ \frac{\sum_{j\in J_{1}}{R^{j}}_{K}}{\min_{j\in J_{1}}{R^{j}}_{K}}+\frac{\sum_{j\in J_{2}}{R^{j}}_{K}}{\max_{j\in J_{2}}{R^{j}}_{K}}\left(\;\mathrm{if}\;j\in J_{1}\;\mathrm{then}\;{R^{j}}_{K}<0,\;\mathrm{if}\;j\in J_{2}\;\mathrm{then}\;{R^{j}}_{K}>0\right)\text{,} \end{array}$$

(3) $$\begin{array}{l} {\mathrm{TopoVal}}_{2}=\\ \frac{\sum_{j\in J_{1}}{R^{j}}_{K}}{\max_{j\in J_{1}}{R^{j}}_{K}}+\frac{\sum_{j\in J_{2}}{R^{j}}_{K}}{\min_{j\in J_{2}}{R^{j}}_{K}}\left(\mathrm{if}\;j\in J_{1}\;\mathrm{then}\;{R^{j}}_{K}>0,\;\mathrm{if}\;j\in J_{2}\;\mathrm{then}\;{R^{j}}_{K}<0\right)\text{.} \end{array}$$

In our study, the ERD/ERS phenomenon was further evaluated using topographic value ( TopoVal for short). In Equation 2, we assume the *i*-th trial to be right-hand MI type and calculate its TopoVal , i.e., $\mathrm{TopoVa}l_{1}$ . Likewise, in Equation 3, we also suppose this trial to be left-hand MI type and compute its TopoVal , i.e., $\mathrm{TopoVa}l_{2}$ .

In Equation 1, for the *i*-th trial belonging to class *c*, if the true class’s TopoVal , i.e., $\mathrm{TopoVa}l_{c}$ , is greater than the opposite class’s TopoVal , it is considered to have normal ERD/ERS characteristic and is given a “good” evaluation. Otherwise, it is thought to have weak ERD/ERS feature and is encouraged with a “fight” hint.

Obviously, if the correctly activated electrodes have deeper color, the topographic map has more apparent ERD/ERS phenomenon. On the contrary, if the wrongly activated electrodes have deeper color, the map has weak ERD/ERS characteristic. The color depth of activated electrodes was measured as follows. In Equations 2 and 3, for all electrodes evoking ERS feature, we calculate $\sum_{j}{R^{j}}_{K}/\max_{j}{R^{j}}_{K}$ , where ${R^{j}}_{K}>0$ . Likewise, for all electrodes triggering ERD characteristic, we compute $\sum_{j}{R^{j}}_{K}/\min_{j}{R^{j}}_{K}$ , where ${R^{j}}_{K}<0$ .

**Figure 5:**
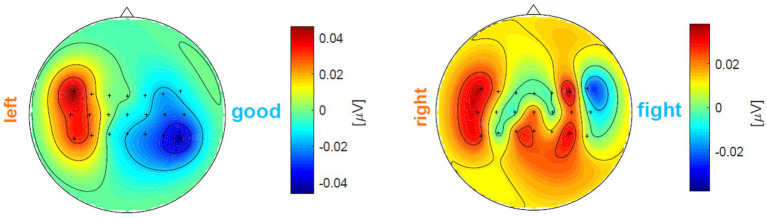
A left-hand MI trial’ prompt feedback and a right-hand MI trial’ prompt feedback.

Let us give two examples to better understand in Figure 5. In the left subfigure, the left-hand MI trial’s TopoVal , i.e., $\mathrm{TopoVa}l_{2}$ , is obviously larger than the opposite class’s TopoVal , i.e., $\mathrm{TopoVa}l_{1}\text{,}$ thus this trial is a good one. However, in the right subfigure, after calculation, the right-hand MI trial’s TopoVal , i.e., $\mathrm{TopoVa}l_{1}$ , is less than $\mathrm{TopoVa}l_{2}$ , which suggests that more electrodes are wrongly activated.

It was noted that such evaluation cannot be translated into a command to retain or abandon the current trial due to the lack of understanding of overall feature distribution.

#### 2.5.3 Signal processing

In our experiment, the same signal processing methods were used in the calibration runs of different sessions since we focused on different paradigms.

We sequentially performed data preprocessing, artifact removal, feature extraction, and classification algorithm after three or six calibration runs.

First, all raw EEG signals from each calibration run were band-pass filtered between 0.1 and 30 Hz with an eighth order Butterworth filter, and notch filtered between 48 and 52 Hz. Each MI training trial was extracted from the time interval between 0.5 and 2.5 s after the arrow visual cue onset.

After preprocessing, if the peak amplitudes of EOG signals captured in the confirmation phase were greater than the threshold of blinking features, the corresponding MI trials would be marked as dissatisfied ones and removed from the training set. Let the size of the remaining training set be $N_{\mathrm{remove}:\mathrm{dis}}$ ( $N_{\mathrm{remove}\_\mathrm{dis}}\leq 20)\text{.}$ Then, the EEG training trials with excessive variance were also deleted using the “Artifact Rejection” function from the bbci toolbox. The procedure of the artifact rejection can be summarized as below.

##### ALGORITHM 2: Remove the training trials with excessive variance

**Input**: the remaining set after removing dissatisfied trials: $TR_{\mathrm{remove}:\mathrm{dis}}=$ ${\left\{X_{i}|X_{i}\in R^{512\times 21}\right\}}_{i=1}^{N_{\mathrm{remove}:\mathrm{dis}}}$ .
**Output:** the final training set: $TR_{\mathrm{final}}={\left\{X_{i}|X_{i}\in R^{512\times 21}\right\}}_{i=1}^{N_{\mathrm{final}}}$ .
Initialize: $N_{\mathrm{final}}=N_{\mathrm{remove}:\mathrm{dis}}$ , $TR_{\mathrm{final}}=TR_{\mathrm{remove}:\mathrm{dis}}$ ;
**
for** i = 1 to $N_{\mathrm{remove}:\mathrm{dis}}$ **do
**
Calculate the variance $\sigma_{i}\left(\sigma_{i}\in R^{1\times 21}\right)$ for the *i*-th trial $X_{i}$ ;
**
end for
**
**
for** j = 1 to 21 **do**
**if** more than 10% of trials have small variances ( <0.5 ) at the *j*-th electrode **then**
**
for** i = 1 to $N_{\mathrm{remove}:\mathrm{dis}}$ **do
**
remove the *j*-th entry of $\sigma_{i}$ and then obtain new variance ${\dot{\sigma }}_{i}\;\left({\dot{\sigma }}_{i}\in R^{1\times N_{e}},\;N_{e}\leq 21\right)$ ;
**
end for
**
**
end if
**
**
end for
**
**
do
**
Concatenate the remaining trials’ ${\dot{\sigma }}_{i}$ and obtain a new vector Var ;
Compute the threshold: $\begin{array}{l} \mathrm{threshold}=\mathrm{percentile}\left(Var,90\right)+3\times \;\\ \left(\mathrm{percentile}\left(Var,90\right)-\mathrm{percentile}\left(Var,10\right)\right) \end{array}$ ;
**for** each trial from $TR_{\mathrm{final}}$ **do
**
**if** its variance is greater than threshold **then
**
Remove that trial from $TR_{\mathrm{final}}$ ;

$N_{\mathrm{final}}=N_{\mathrm{final}}-1$ ;
**
end if
**
**
end for
**
**if** no trial is removed due to excessive variance **then**
break;
**
end if
**
**while** (true)

In Algorithm 2, $\mathrm{percentile}\left(Var,t\right)$ returns the t -th percentile of Var . For example, $\mathrm{percentile}\left(Var,50\right)$ returns the median of Var .

Then, Riemannian alignment (RA) (Zanini et al., 2018; He and Wu, 2019; Xu et al., 2021) and common spatial pattern (CSP) method (Ramoser et al., 2000) were successively executed to extract valuable feature vectors. More details can be found as below.

For each calibration run, the Riemannian mean $M_{R}$ can be defined as


(4) $$M_{R}=\underset{M}{\mathrm{argmin}}\sum_{i=1}^{N_{\mathrm{final}}}{\left(||\log\left({P_{i}}^{-1}M\right)||{\;}_{F}\right)}^{2}\text{,}$$

where $P_{i}$ is the covariance matrix of $X_{i}$ , and $||.||{\;}_{F}$ performs the Frobenius normalization operation.

The *i*-th trial $X_{i}$ can be aligned using the Riemannian mean $M_{R}$ to shorten the inter-run differences as


(5) $${\tilde{X}}_{i}=X_{i}{M_{R}}^{\left(-1/2\right)}\text{.}$$

After performing RA for each calibration run, the CSP feature extraction algorithm was executed. Let ${\tilde{P}}_{i}$ be covariance matrix of the *i*-th aligned trial ${\tilde{X}}_{i}$ from three or six training sets. Then, the average covariance matrix from class *c* can be computed as


(6) $$P_{c}=\frac{1}{N_{c}}\sum_{i=1}^{N_{c}}{\tilde{P}}_{i}\text{,}$$

where $N_{c}$ is the number of all training trials from class *c* (*c* ∈ [1,2]).

To maximize the discriminability of two populations of EEG signals, the Rayleigh quotient can be maximized by


(7) $$W_{p}=\underset{\omega }{\mathrm{argmax}}\frac{\omega^{T}P_{1}\omega }{\omega^{T}P_{2}\omega }\text{,}$$

where $W_{p}$ is the projection matrix and ${\left(.\right)}^{T}$ is the transpose operation. In Equations 4–7, we obtained the CSP projection matrix after performing RA and CSP methods. In our study, the CSP spatial filters $W_{s}$ were extracted from the first three rows and the last three rows of $W_{p}$ . The first three spatial filters can maximize the variance of class 1 while minimizing the variance of class 2. Likewise, the last three spatial filters can yield the high variance of class 2 and the low variance of class 1. Then, the six-dimensional feature vector $F_{i}$ was the logarithm of the variance of ${\tilde{X}}_{i}$ after projection onto $W_{s}$ .

Finally, these low-dimensional CSP feature vectors from the three or six calibration runs were used to construct the subject-specific linear discriminant analysis (LDA) hyperplane (Lotte et al., 2007). The normal vector w of the hyperplane can be defined by


(8) $$w={\left(\frac{\mathrm{cov}\left(F_{1}\right)+\mathrm{cov}\left(F_{2}\right)}{2}\right)}^{-1}{\left({\bar{F}}_{1}-{\bar{F}}_{2}\right)}^{T}\text{,}$$

where $F_{c}$ is the *c*-th class feature set with $N_{c}$ size, $\mathrm{cov}\left(F_{c}\right)$ computes the covariance matrix of $F_{c}$ , and ${\bar{F}}_{c}$ is the mean of the *c*-th class feature set.

The bias of the hyperplane can be given by


(9) $$b=-\frac{1}{2}\left({\bar{F}}_{1}+{\bar{F}}_{2}\right)w\text{.}$$

In Equations 8 and 9, the normal vector and bias of the LDA hyperplane can be obtained. Then, the LDA model $f\left(F_{i}\right)=\mathrm{sign}\left(wF_{i}+b\right)$ can be used to judge the class of the Feature $F_{i}$ .

### 2.6 Run evaluation

**Figure 6:**
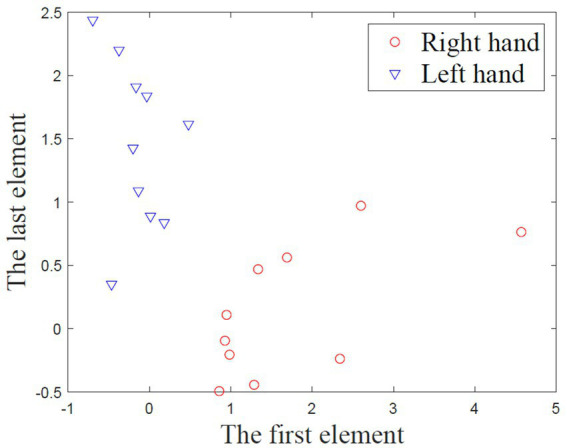
The example of CSP feature distribution.

A run evaluation was provided after each calibration run of trial-feedback session. For the trial-feedback session, a run evaluation, in which the CSP feature distribution was visualized and quantified, was conducted after each calibration run. As mentioned above, each aligned MI training trial can be transformed into a six-dimensional CSP feature. Then, the first and last elements of all CSP features, which were optimal to discriminate two populations of EEG trials, were projected into a two-dimensional screen. Figure 6 shows an example of CSP feature distribution.

The CSP feature distribution can be quantified by class distance (*CD* for short). Motivated by the domain transferability in (Zhang and Wu, 2020), we adopted the within-class matrix $S_{w}$ and between-class matrix $S_{b}$ to measure the aggregating ability of each class and the discriminating ability of binary classes, respectively. In Equations 10–12 $S_{w}$ , $S_{b}$ , and *CD* can be calculated as follows:


(10) $$S_{w}=\sum_{c=1}^{2}F_{c}\left(I-\frac{1_{N_{c}}}{N_{c}}\right){F_{c}}^{T}\text{,}$$

(11) $$S_{b}=\sum_{c=1}^{2}N_{c}\left({\bar{F}}_{c}-\bar{F}\right){\left({\bar{F}}_{c}-\bar{F}\right)}^{T}\text{,}$$

(12) $$CD=\|S_{b}\|{\;}_{1}/\|S_{w}\|{\;}_{1}\text{,}$$

where $F_{c}$ denotes the *c*-class CSP feature set from one calibration run, $N_{c}$ is the size of $F_{c}$ , I means an $N_{c}$ -dimensional identity matrix, $1_{N_{c}}\in R^{N_{c}\times N_{c}}$ is an all-one matrix, ${\bar{F}}_{c}$ and $\bar{F}$ denote the mean of the *c*-class feature set and the mean of all feature sets, respectively, $||.||{\;}_{1}$ performs the normalization computation.

### 2.7 Testing run

**Figure 7:**
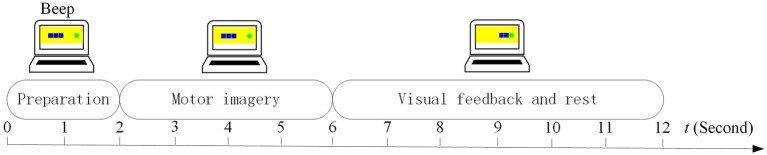
Timing schedule of a trial in the testing run.

With the aim of investigating the effect of the size of training set, two models were trained using the trials from the first three and six calibration runs, respectively. They were separately applied to the first two and last two testing runs. Five testing trials per MI task type were performed in each testing run. The timing schedule of a trial in the testing run is shown in the Figure 7.

At the beginning of a trial, a green food randomly appeared on the left or right side of the screen to promote the subject to make preparation. After 2 s and a short beep, the subject concentrated on performing the assigned MI task for 4 s. During the phase of visual feedback and rest, the 4-s testing trial was processed as follows. First, the EEG testing trial was band-pass filtered between 0.1 and 30 Hz with an eighth order Butterworth filter, notch filtered between 48 and 52 Hz, and segmented between 0.5 and 2.5 s from 4-s data epoch. Then, this trial was aligned using all training trials’ Riemannian mean. Next, the aligned trial was converted into corresponding CSP feature vector using the spatial filters mentioned above. Finally, the feature vector was input to the LDA model and translated into a command that drove the blue snake to move left or right.

### 2.8 Questionnaire

In our study, we designed different questionnaires to strengthen subject’s involvement and feedback. Each subject should fill in questionnaires regarding individual pre/post-experimental status after completing all MI tasks. For the calibration process of the trial-feedback session, the post-questionnaire was provided to the subject prior to the run evaluation. Except for the expected *CA*, the number of blinks, and the number of naps, most pre/post-experimental status were quantified with a Likert scale ranging from 1 to 5. Table 1 lists the questionnaire in detail.

**Table 1:** Questionnaire about individual pre/post-experimental status.

Occasion	Question and its possible values
Before the first calibration run	Sleep duration(h = hours): 1 (>8 h), 2 (7 ~ 8 h), 3 (6 ~ 7 h), 4 (5 ~ 6 h), 5 (<4 h)
	Did you drink coffee? 1 (no), 5 (yes)
	Did you drink alcohol? 1 (no), 5 (yes)
	How do you feel now? 1 (very good), 2 (good), 3 (so-so), 4 (bad), 5 (very bad)
	Healthy condition: 1 (very good), 2 (good), 3 (so-so), 4 (bad), 5 (very bad)
	The expected *CA*: 0% ~ 100%
After each calibration run	How do you feel now? 1 (very good), 2 (good), 3 (so-so), 4 (bad), 5 (very bad)
	Attention state: 1 (very high), 2 (high), 3 (so-so), 4 (low), 5 (very low)
	Is MI training easy? 1 (very easy), 2 (easy), 3 (so-so), 4 (hard), 5 (very hard)
	Number of blinks: <=number of trials
After each testing run	Attention state: 1 (very high), 2 (high), 3 (so-so), 4 (low), 5 (very low)
	Is MI testing easy? 1 (very easy), 2 (easy), 3 (so-so), 4 (hard), 5 (very hard)
	Number of naps: <=number of trials

## 3 Experimental results

In our study, we aimed to improve the MI training paradigm for subject’s self-modulation. In the proposed trial-feedback paradigm, we only provided the on-line qualitative evaluation for each MI training trial, rather than the on-line quantitative evaluation. Thus, it was difficult to quantitatively measure the effect of self-modulation in real time. In the following, various metrics can reflect the result of self-modulation.

For the non-feedback and trial-feedback sessions, we first evaluated each subject’ MI-BCI performance in the training process. Two metrics were used to assess the subject’s ability to distinguish the different MI task types: class distance (*CD*) and off-line classification accuracy (*CA*). The *CD* value was obtained in the feature extraction phase, whereas the *CA* value was calculated in the subsequent feature learning phase. Moreover, the first and last CSP spatial filters were utilized to show the subject’s ability to control the cerebral rhythm. Then, we testified the effectiveness of our proposed paradigm in the testing procedure using on-line classification accuracy (*CA*). Finally, a subjective report collected in the training and testing runs was shown to reveal the subject’s feelings on different sessions.

### 3.1 Training process evaluation

#### 3.1.1 Class distance results

In Table 2, we present 10 subjects’ *CD* values in the calibration processes of different sessions to measure the distinctiveness between the left-hand and right-hand CSP features from six calibration runs. The superscript of *CD* represents certain session (non-feedback and trial-feedback are abbreviated as *Non* and *Trial*, respectively). And the subscript *cal* of *CD* means the calibration process. It was noted that the $CD_{\mathrm{cal}}^{Non}$ values were not given to the subjects and were calculated just for comparison. Ten subjects were further divided into the trained and untrained groups to compare the two groups’ performance across different paradigms. The bold-faced number shows the best result.

**Table 2:** Ten subjects’ *CD* values in the calibration processes of different sessions.

	Trained						Untrained						All
	S1	S2	S3	S4	S5	Mean	S6	S7	S8	S9	S10	Mean	Mean
$CD_{\mathrm{cal}}^{Non}$	0.46	0.89	**1.19**	0.46	1.18	0.84	**0.89**	**0.78**	0.50	0.56	**0.98**	**0.74**	**0.79**
$CD_{\mathrm{cal}}^{\mathrm{Trial}}$	**0.51**	**1.26**	0.69	**0.59**	**1.31**	**0.87**	0.46	0.49	**0.88**	**0.60**	0.78	0.64	0.76

The bold values mean the better CD results.

As shown in Table 2, the trained subjects performed better than the untrained subjects on average in the calibration processes of two sessions, indicating the usefulness of MI experience. In addition, the *CD* differences between the calibration processes of different sessions were not significant according to a paired samples T-Test (*p* = 0.7520). The average *CD* value over 10 subjects for the non-feedback paradigm was slightly higher than that for the trial-feedback paradigm. However, four out of five trained subjects obtained greater *CD* values for the trial-feedback paradigm than for the non-feedback paradigm, three out of five untrained subjects yielded better *CD* values for the non-feedback paradigm than for the trial-feedback paradigm.

In terms of the *CD* metric, the trial-feedback paradigm did not exhibit obvious advantage over the non-feedback paradigm. In our opinion, the *CD* metric can only reflect the overall CSP feature distribution, whereas the off-line *CA* value can further evaluate the differences between two classes of CSP features.

#### 3.1.2 Off-line classification accuracy

The off-line *CA* was computed by adopting 10 × 10-fold cross validation on available training data from six calibration runs for each subject.

**Figure 8:**
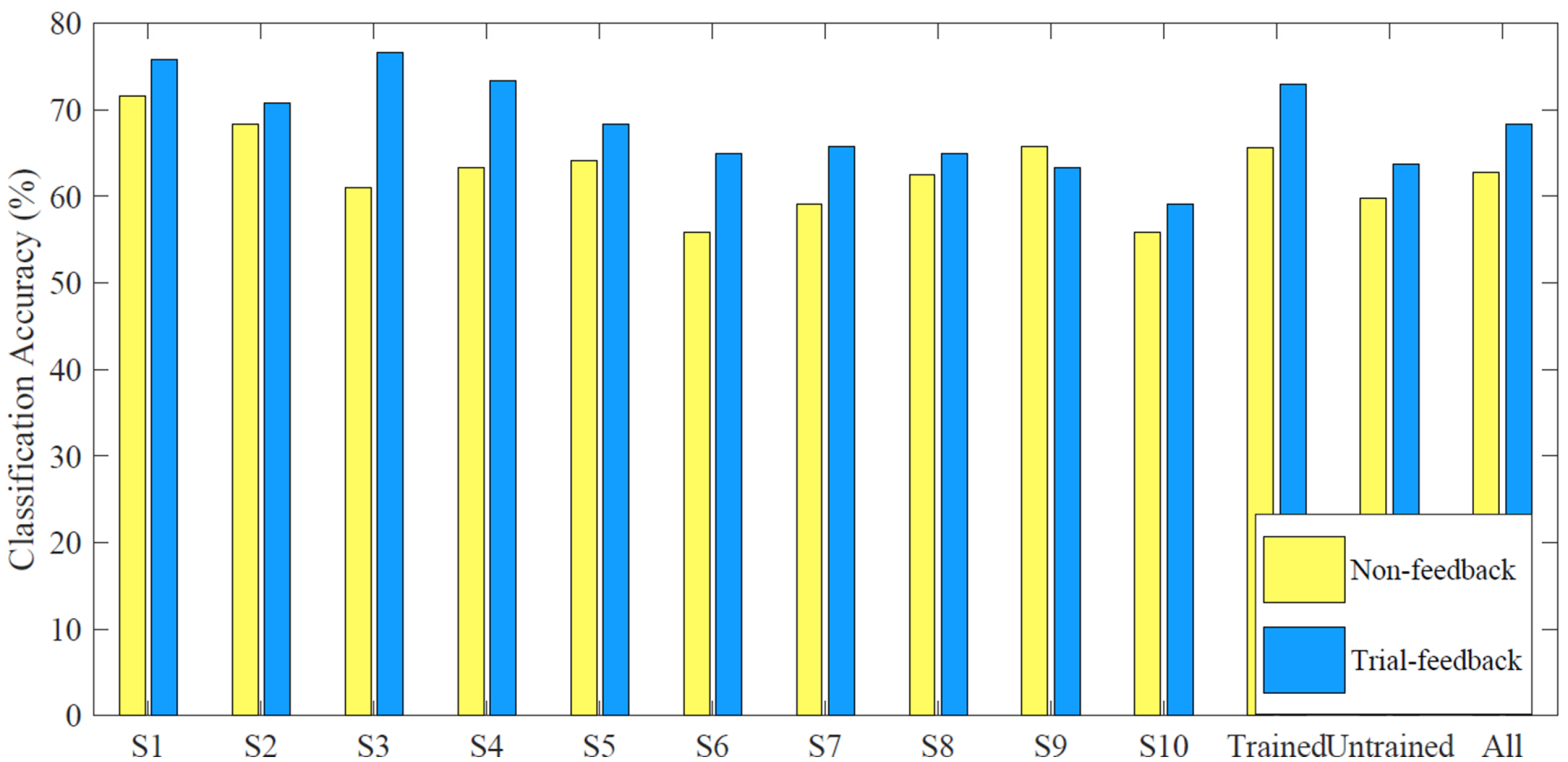
Average off-line *CA* values of each subject, each group, and all subjects in the calibration processes of different sessions.

The average off-line *CA* values of each subject, each group, and all subjects in the calibration processes of different sessions are shown in Figure 8.

Results showed that only subject S9 performed slightly better for the non-feedback paradigm than for the trial-feedback one. Subjects S3, S4, and S6 achieved the greater improvement via trial-feedback paradigm (increased by over 10%). It indicated that the trial-feedback paradigm was helpful for most subjects to distinguish different MI tasks. A paired T-Test showed that the average off-line *CA* values across 10 subjects were significantly different between two paradigms (*p* = 0.0068). Compared to the slight *CD* differences between different paradigms, the obvious off-line *CA* differences were more valuable since the two-class CSP features were further recognized by the classifier.

In addition, the improvement (trial-feedback vs. non-feedback) in the trained group was much better than that in the untrained group (11.11% vs. 6.42%). Moreover, the average off-line *CA* value of the trained group for the non-feedback paradigm was slightly higher than that of the untrained group for the trial-feedback paradigm, further suggesting the usefulness of MI-BCI experience. Nevertheless, the average off-line *CA* value of the trained group for the trial-feedback paradigm was not high enough. The possible explanation was the small-sized training set.

#### 3.1.3 Visualization of spatial filters

The *CD* and off-line *CA* metrics can only show the subject’s ability to distinguish different MI tasks, whereas the visualization of CSP spatial filters can further reflect the subject’s ability to perform MI tasks.

**Figure 9:**
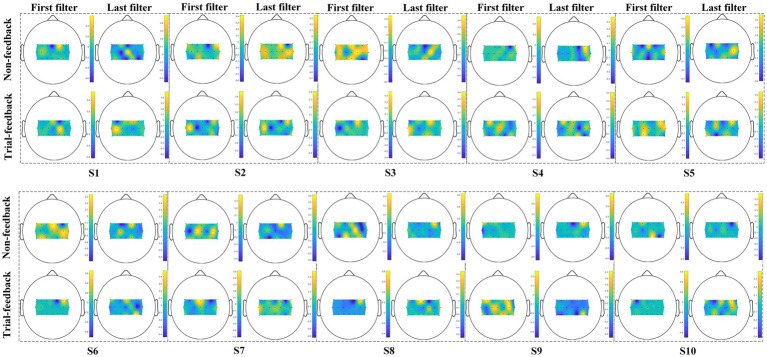
Ten subjects’ topographic maps of the first and last spatial filters under the two different paradigms.

Since the CSP spatial filters can improve the SNR of EEG data, they are useful in the neurophysiological understanding of ERD/ERS phenomenon (Ramoser et al., 2000). Among all spatial filters, the first and last filters are the most representative ones. In Figure 9, 10 subjects’ first and last spatial filters extracted from the training trials of the two different paradigms are topographically mapped onto a scalp using the regularized CSP (RCSP) toolbox (Lotte and Guan, 2011).

As mentioned above, class 1 and class 2 correspond to the right-hand and left-hand MI tasks, respectively. Therefore, the first filter displays the ERS phenomenon over the right hemisphere and the ERD feature over the left cortex. On the contrary, the last filter associates with the ERD/ERS phenomenon over the opposite sensorimotor areas.

As shown in Figure 9, for subjects S1, S2, S3, S6, S7, and S8, the first and last filters appear as messier in the non-feedback paradigm than in the trial-feedback paradigm. In general, the ERS phenomenon seems to be more obvious due to the brighter color. Results showed that, for subjects S1, S3, S7, and S8, the CSP filters obtained the apparent ERS characteristic in the trial-feedback paradigm, with large weights over the expected sensorimotor areas. However, the remaining subjects did not show clear ERD/ERS phenomenon in the two paradigms.

### 3.2 Testing process evaluation

**Figure 10:**
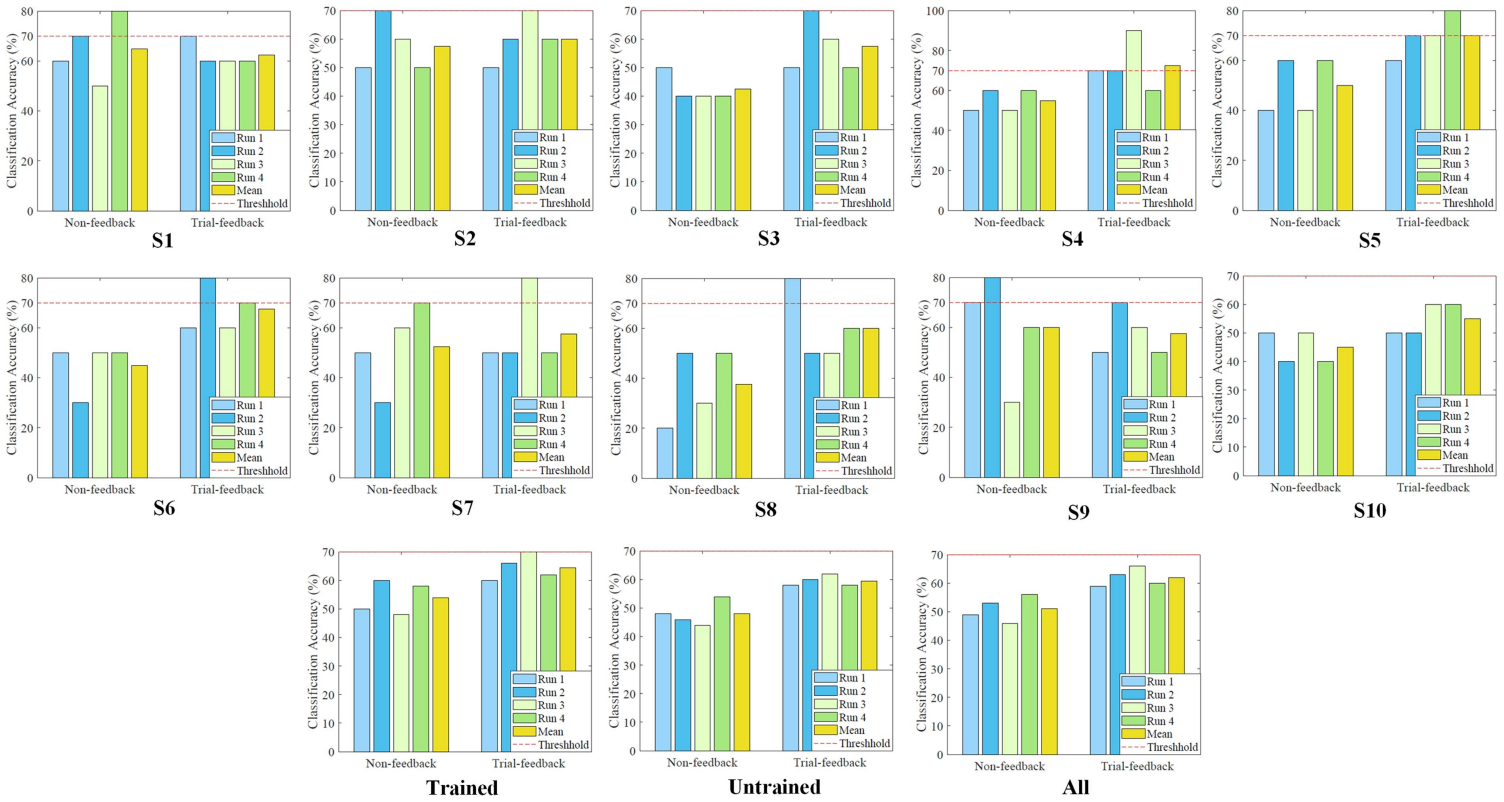
The on-line *CA* values of the four testing runs from different sessions, and their averages for each subject, each group, and all subjects.

In the testing procedure, a simple brain-controlled Belly-Worship snake game was designed to evaluate the effectiveness of different MI paradigms. To reduce the subject’s burden, only four testing runs of 10 MI trials each were required to be executed. Two different models trained on the first three and six calibration runs were separately applied to the first two and last two testing runs. The on-line *CA* was acquired by counting the correctly identified testing data from each testing run. The on-line *CA* values of the four testing runs from the different sessions and their averages for each subject, each group, and all subjects are shown in Figure 10. A red dashed line represents the benchmark *CA* of 70%.

As depicted in Figure 10, in the first two testing runs, the benchmark *CA* has been reached or exceeded five and eight times for the non-feedback and trial-feedback sessions, respectively, while in the last two testing runs, the classification performance is equal to or greater than this threshold two and six times for the non-feedback and trial-feedback sessions, respectively. Unfortunately, about a quarter [(5 + 8 + 2 + 6 = 21)/(2 sessions × 4 runs × 10 subjects = 80) = 26.25%] of testing runs met BCI benchmark *CA.* As shown in the last subfigure, the average on-line *CA* value of the first two runs in the two sessions [(49% + 53% + 59% + 63%)/4 = 56%] is slightly lower than that of the last two testing runs in the two sessions [(46% + 56% + 66% + 60%)/4 = 57%]. Furthermore, most subjects could not always keep good mental states. Only subjects S5 and S10 obtained growing on-line *CA* values in the four testing runs of the trial-feedback session.

Besides, in terms of different paradigms, only subjects S1 and S9 obtained the better average *CA* values for the non-feedback session whereas the other subjects performed better for the trial-feedback session. On average, the trial-feedback paradigm showed its superiority in the testing process. In the trained group, the average on-line *CA* of the non-feedback session (54%) was lower than that of the trial-feedback session (64.5%). In the untrained group, the non-feedback session had the average on-line *CA* (48%) merely around the chance level, while the trial-feedback session obtained a significant improvement of 24%. The *CA* differences between the 40 (4runs × 10subjects) testing runs from different sessions were obvious according to the paired samples T-Test (non-feedback vs. trial-feedback: *p* < 0.005).

The Pearson correlations between the average off-line *CA* and the average on-line *CA* for 10 subjects in the non-feedback and trial-feedback sessions were (*r* = 0.7239, *p* = 0.0179, *N* = 10) and (*r* = 0.3246, *p* = 0.3602, *N* = 10), respectively.

### 3.3 Subjective report

Apart from those objective metrics, we also included a subjective report that reflected 10 subjects’ feelings on different processes of different sessions. In our study, we did not investigate the relationship between the individual pre-experimental status and the MI-BCI performance. Instead, we focused on individual replies to the post-experimental status except for the number of blinks and the number of naps.

A questionnaire (*Qn* for short) value is the average of all post-experimental status mentioned above. For example, subject S1’s $Qn_{\mathrm{cal}}^{Non}$ is the average of *Qn* values of all calibration runs in the non-feedback session. To study subjective feelings, 10 subjects’ *Qn* values in the different processes of different sessions are shown in Table 3.

**Table 3:** Ten subjects’ *Qn* values in the calibration and testing processes of different sessions.

	Trained						Untrained						All
	S1	S2	S3	S4	S5	Mean	S6	S7	S8	S9	S10	Mean	Mean
$Qn_{\mathrm{cal}}^{Non}$	3.67	**3.00**	**3.00**	**3.83**	**3.00**	3.30	2.28	**2.56**	**3.00**	2.06	**3.00**	2.58	2.94
$Qn_{\mathrm{cal}}^{\mathrm{Trial}}$	**3.00**	**3.00**	**3.00**	**3.83**	**3.00**	**3.17**	**2.00**	2.72	**3.00**	**1.44**	**3.00**	**2.43**	**2.80**
$Qn_{\mathrm{test}}^{Non}$	3.25	**3.25**	3.58	**4.25**	**3.00**	3.47	2.58	2.75	**3.00**	2.00	**3.00**	2.67	3.07
$Qn_{\mathrm{test}}^{\mathrm{Trial}}$	**3.00**	**3.25**	**3.08**	4.50	**3.00**	**3.37**	**2.00**	**2.67**	**3.00**	**1.33**	**3.00**	**2.40**	**2.88**

The bold values represent the better Qn values in the certain process of different sessions.

All post-experimental status related to the *Qn* value are based on a 1–5 Likert scale to indicate very good, good, so-so, bad, or very bad status. Obviously, the smaller *Qn* value reflects the better post-experimental status.

As listed in Table 3, in terms of the average *Qn* value, a paired *T*-Test showed that there were not apparent differences between different processes of different sessions across 10 subjects ( $Qn_{\mathrm{cal}}^{Non}$ vs. $Qn_{\mathrm{cal}}^{\mathrm{Trial}}$ : *p* = 0.1540, $Qn_{\mathrm{test}}^{Non}$ vs. $Qn_{\mathrm{test}}^{\mathrm{Trial}}$ : *p* = 0.0894). However, the trial-feedback session took an advantage over the non-feedback session in the trained and untrained groups. Compared with the calibration and testing processes of the non-feedback session, subjects S1, S3, S6, and S9 obtained the lower *Qn* values in the corresponding processes of the trial-feedback session.

Interestingly, for different sessions, the average *Qn* values in the untrained group were always lower than those in the trained group. The untrained subjects S6, S7, and S9 expressed their good post-experimental status (*Qn* < 3) in the different processes of different sessions. However, no trained subjects’ *Qn* values were less than 3. The possible explanation was that the untrained subjects felt more relaxed than the trained subjects. Overall, the average *Qn* values of the calibration runs were usually less than those of the testing runs, indicating that the subjects felt more nervous in real-time BCI application.

## 4 Discussion

### 4.1 Superiorities

In our study, we aimed at improving the MI training procedure to help the subject modulate the brain activity. To investigate whether the subject was executing MI tasks well, based on the literature from Hwang et al. (2009), the topographic map closely related to the ERD/ERS phenomenon was selected as an important part of our prompt feedback. However, different from their study, in our trial-feedback paradigm, the optimal map was chosen from three maps with different temporal segments for real-time display depending on the number of correctly activated electrodes. Additionally, an objective evaluation of the current training trial was conducted based on the ERD/ERS phenomenon, which helped to understand the complex map. Bai et al. (2014) demonstrated the ERD/ERS characteristics of four electrodes on the four panels. However, we showed the ERD/ERS features of more electrodes through one topographic map, providing a simpler and richer perspective. Duan et al. (2021) provided an on-line data visualization, while we visualized and quantified a feature distribution after each calibration run of the trial-feedback session, facilitating the comparison between different calibration runs. Unlike Mladenović et al. (2022), our prompt feedback did not rely on the classification output, making it applicable from the beginning of calibration process. In contrast to Darvishi et al. (2017) and so on, our training protocol was easy to implement without using expensive external devices.

In our work, we focused on comparing the trial-feedback paradigm with the non-feedback paradigm. Such comparison was meaningful since the non-feedback paradigm was similar to the commonly used traditional MI paradigm. Thus, we designed the trial-feedback session and the non-feedback session. Each session included one EOG run, six calibration runs to use the paradigm, and four testing runs to evaluate the paradigm. Moreover, 10 subjects were divided into the trained and untrained groups to investigate the role of MI experience.

As mentioned above, the subjects could explore their own imagination movements. For the non-feedback paradigm, the subjects knew nothing about the quality of their MI training trials during and after the calibration run. And they reported that they rarely adjusted their MI behaviour during the training process due to lack of feedback. However, for the trial-feedback paradigm, the subjects knew whether their current trial conformed to the ERD/ERS phenomenon via the on-line topographic map and corresponding evaluation. So, they could remain or modulate their imagination movements after receiving a “good” hint or a “fight” hint. They could adjust their mental strategies by improving their concentration or the amplitudes of imagination movements. However, the subjects might feel frustrated by a “fight” hint. Thus, all subjects were encouraged to calmly face the “fight” evaluation. In the following, various objective and subjective metrics were used to assess the subject’s MI-BCI skills in the different processes of different sessions for investigating the role of feedback and the result of self-modulation.

To study the impact of different paradigms on the calibration process, we first analysed the *CD* metric, the off-line *CA* metric, and the CSP spatial filter visualization as follows.

The *CD* and off-line *CA* values were used to evaluate the subject’s MI-BCI ability to distinguish the different MI tasks during the calibration runs. The CSP feature distribution was quantified by calculating the *CD* value. In Table 2, six out of 10 subjects obtained better *CD* values in the trial-feedback paradigm than in the non-feedback paradigm, suggesting the usefulness of the prompt feedback and the run evaluation. However, the *CD* differences between the calibration processes of two sessions were not obvious. The possible reasons were the following. First, the subjects were always instructed how to imagine the movements of limbs rather than how to make the two-class MI trials far from each other. Secondly, the CSP feature distribution associated with the *CD* value was only given after the calibration run of the trial-feedback session, resulting in delayed self-modulation of the subjects. Additionally, in Figure 8, nine out of 10 subjects achieved greater average off-line *CA* values in the trial-feedback paradigm than in the non-feedback paradigm. Compared to the slight *CD* differences, the obvious off-line *CA* differences may be attributable to the effective classifier being able to recognize and amplify the differences between two categories of CSP features. Moreover, the trained group exhibited better average *CD* and off-line *CA* values in the trial-feedback paradigm than in the non-feedback paradigm. Nevertheless, on average, the untrained group achieved higher *CD* value and lower off-line *CA* value in the non-feedback paradigm than in the trial-feedback paradigm. We inferred that the artifacts from the training sets of the untrained subjects might increase the *CD* values and decrease the off-line *CA* values. Overall, the *CD* and off-line *CA* results showed that subjects could adjust themselves well in different MI tasks using the prompt feedback and the run evaluation.

The CSP spatial filter visualization was used to assess the subject’s MI-BCI ability to perform the assigned MI tasks. The *CD* and off-line *CA* metrics can only reflect the aggregating extent of each class and the discriminating extent of two classes, while the topographic maps of the first and last CSP spatial filters can present the neurophysiological evidence of the subject’s imagination movements. In Figure 9, three trained subjects (S1, S2, and S3) and three untrained subjects (S6, S7, and S8) obtained messier maps of filters in the non-feedback paradigm than in the trial-feedback paradigm, suggesting that some trained and untrained subjects cannot execute the MI tasks well without real-time feedback. Furthermore, two trained subjects (S1 and S3) and two untrained subjects (S7 and S8) exhibited the obvious ERS feature in the trial-feedback paradigm, indicating the prompt feedback was useful in improving the subject’s self-modulation. Interestingly, in Figure 8, for the trial-feedback paradigm, these four subjects (S1, S3, S7, and S8) obtained the higher off-line *CA* values in their trained or untrained group, demonstrating a positive relationship between the ability to perform the MI tasks and the ability to distinguish different tasks. However, owing to the subject’s BCI illiteracy behaviour, the other subjects did not acquire obvious ERD/ERS feature in the two paradigms. In terms of the CSP filter visualization, our trial-feedback paradigm did not show significant superiority over the non-feedback one. Nevertheless, the former was helpful for most subjects to improve the ERD/ERS phenomenon. Thus, the subjects could achieve positive self-modulation with the help of prompt feedback.

To our knowledge, many similar studies have only designed the calibration process (Abu-Rmileh et al., 2019; Duan et al., 2021; Mladenović et al., 2022). In our study, additional testing process was further utilized to investigate the effectiveness of training process. Next, we discussed the subjects’ performance in the testing runs of different sessions using the on-line *CA* values. Due to the use of the same signal processing algorithms, the performance differences were mainly correlated with the quality of the training and testing samples, as well as their similarities. We found that the testing process had a strong correlation with the training procession due to the positive Pearson correlations between the average on-line *CA* values and the average off-line *CA* values across 10 subjects. The on-line *CA* differences between the testing runs of different sessions were statistically significant. Eight out of 10 subjects achieved higher on-line *CA* values in the trial-feedback session than in the non-feedback session on average. Both the trained and untrained groups achieved great improvements in the trial-feedback session. It indicated that our improved training protocol could foster the subjects’ performance in real-time BCI application. However, the subjects could not always perform MI tasks well in the different testing runs of the two sessions. Subjects reported that the consecutive successful or unsuccessful MI tasks might promote or degenerate their subsequent experimental status. Thus, the subject’s unsteady mental state was the major reason for instable on-line *CA* values. Besides, EEG non-stationarity, muscular artifacts, environmental noises might cause inter-run variability. Additionally, after a short calibration process, some subjects can sometimes achieve the benchmark *CA* of 70% in the first two testing runs. We deduced that those testing samples might close to the training samples with the same class. Nevertheless, the subjects performed better in the last two testing runs than in the first two testing runs on average, indicating the necessity of more training trials. In our study, we always mentioned that the subjects could modulate their brain activities during the calibration process of the trial-feedback session. In fact, under the prompt of classification output, the subjects could also adjust their neural rhythm during the testing processes of different sessions. However, the subject’s self-modulation in the testing process could not improve the robustness of the classifier. Therefore, it is of importance to enhance the quality of MI training trials through subject’s self-adjustment.

Then, we analyzed the subjective metric *Qn* which revealed each subject’s the post-experimental status after different processes of different sessions. Lower *Qn* value represented more positive experimental feeling of the subject. In Table 3, the trial-feedback session shows the lower *Qn* values than the non-feedback session on average from the calibration procedure to the testing procedure, indicating that subjects prefer our improved training protocol and subsequent testing results. However, the *Qn* differences between the calibration procedures of different sessions were not significantly obvious than those between the corresponding testing procedures according to the paired T-Test. The possible reasons were the following. First, during the training procedure, for the non-feedback paradigm, without any feedback, the subjects were unaware of the quality of their MI tasks, so most subjects had neutral attitudes, whereas for the trial-feedback paradigm, the subjects timely received a good or bad feedback on their current MI trials, resulting in their emotional fluctuations. Secondly, although the two sessions had the same testing paradigm and signal processing algorithms, the effective training in the trial-feedback session helped to increase the on-line *CA* values of corresponding testing process and thus promoted the positive feelings of the subjects. Four out of 10 subjects showed the lower *Qn* values in the calibration and testing processes of the trial-feedback session than in the corresponding processes of the non-feedback session, further indicating a close correlation between the calibration and testing processes.

In summary, the experimental results showed that our trial-feedback paradigm was superior to the non-feedback paradigm. With the help of prompt feedback and run evaluation, self-modulation was effective for most subjects. Moreover, the trained subjects can execute the assigned MI tasks better than the untrained subjects.

### 4.2 Limitations

In our experimental design, the following limitations will be improved in our follow-up work.

First, only 10 subjects were involved in our experiments, thus our study was still preliminary. More subjects will be included in our experiments to ensure the generalizability of our training protocol. Although five subjects had MI experience, they were far from experts. Their training effects still had reference significance. Nonetheless, more non-experienced subjects will be involved to better evaluate the impact of our training protocol on improving the MI-BCI performance. Besides, only one female subject participated our experiments. It will be beneficial to recruit more females to balance the gender distribution and investigate gender-related differences in the MI-BCI results. In a word, to promote the MI-BCI system out of laboratory, more inexperienced subjects and female subjects will be recruited in the future to collect more opinions on the different paradigms, find the optimal imagination movements that match the ERD/ERS features, and analyze more general problems.

Secondly, our prompt feedback and run evaluation should be improved. In our training protocol, the prompt feedback only evaluated the current training trial’s ERD/ERS feature but did not show whether the current trial was close to or far away from the existing trials with the same class. In contrast, Duan et al. (2021) presented a real-time EEG data distribution in Riemannian geometry but did not utilize the ERD/ERS feature of MI-BCI. Thus, the combination of on-line global feature distribution and real-time ERD/ERS visualization will be considered. In addition, some novices reflected that the topographic map was abstract, but the objective evaluation was clear. Furthermore, some experienced subjects suggested that we should add quantitative evaluation to better understand current trial. Therefore, the topographic map closely related to the ERD/ERS feature may be quantified and replaced by a smiling face or a crying face with varying degrees in our future study. As for the run evaluation, although a global CSP feature distribution was shown on the two-dimensional screen after each calibration run, such distribution always looked messy due to BCI illiteracy and CSP features’ dimensionality reduction, thereby causing the negative experience for the subject. To make the distribution clearer, we will accumulate MI training trials after each calibration run and use existing MI training trials to generate the CSP feature distribution. In short, more research will be explored for informative, user-friendly real-time feedback and run evaluation.

Finally, our signal processing algorithms should be also improved. In our study, the optimal topographic map was selected from three maps with different time intervals during the calibration runs of trial-feedback session. However, the time interval between 0.5 and 2.5 s was set by default during the corresponding testing runs. In future, the time interval with best ERD/ERS phenomenon will be automatically chosen in the testing procedure. Furthermore, since the subjects’ MI-BCI abilities might fluctuate throughout the session due to their unstable mental and fatigue status, the classifier should be regularly updated to guide the subjects in adjusting their experimental status. She et al. (2019) and Liu et al. (2020) pointed out that the semi-supervised machine learning algorithms can foster the subject learning capacities. Thus, in future, we will explore adaptive semi-supervised machine learning methods by simultaneously utilizing the valuable labeled and unlabeled samples.

## 5 Conclusion

It is well known that MI is a complex mental task owing to the lack of natural control. Therefore, we design the trial-feedback paradigm to assist the subjects to better perform MI tasks using prompt feedback. In the trial-feedback paradigm, we explore a topographic map and its objective evaluation to visualize and evaluate the subject’s MI-BCI skill in real time. Moreover, we visualize and quantify the CSP feature distribution to help the subject understand his/her ability to distinguish the different MI tasks. The non-feedback paradigm is introduced for comparison. To observe the subject’s MI-BCI performance, we include the calibration and testing runs in different sessions. Experimental results demonstrated that our trial-feedback paradigm can help subjects modulate their brain rhythm for better ERD/ERS features and higher classification performance.

## Data Availability

The raw data supporting the conclusions of this article will be made available by the authors, without undue reservation.

## References

[ref1] Abu-Rmileh A. Zakkay E. Shmuelof L. Shriki O. (2019). Co-adaptive training improves efficacy of a multi-day EEG-based motor imagery BCI training. Front. Hum. Neurosci. 13:362. doi: 10.3389/fnhum.2019.00362, PMID: 31680914 PMC6802491

[ref2] Achanccaray D. Izumi S. I. Hayashibe M. (2021). Visual-electrotactile stimulation feedback to improve immersive brain-computer interface based on hand motor imagery. Comput. Intell. Neurosci. 2021, 1–13. doi: 10.1155/2021/8832686

[ref3] Ahn M. Cho H. Ahn S. Jun S. C. (2018). User’s self-prediction of performance in motor imagery brain–computer interface. Front. Hum. Neurosci. 12:59. doi: 10.3389/fnhum.2018.00059, PMID: 29497370 PMC5818431

[ref4] Bai O. Huang D. Fei D. Y. Kunz R. (2014). Effect of real-time cortical feedback in motor imagery-based mental practice training. Neuro Rehabil. 34, 355–363. doi: 10.3233/NRE-131039 24401829

[ref5] Choi J. W. Huh S. Jo S. (2020). Improving performance in motor imagery BCI-based control applications via virtually embodied feedback. Comput. Biol. Med. 127:104079. doi: 10.1016/j.compbiomed.2020.104079, PMID: 33126130

[ref6] Darvishi S. Gharabaghi A. Boulay C. B. Ridding M. C. Abbott D. Baumert M. (2017). Proprioceptive feedback facilitates motor imagery-related operant learning of sensorimotor β-band modulation. Front. Neurosci. 11:60. doi: 10.3389/fnins.2017.00060, PMID: 28232788 PMC5299002

[ref7] Duan X. Xie S. Xie X. Obermayer K. Cui Y. Wang Z. (2021). An online data visualization feedback protocol for motor imagery-based BCI training. Front. Hum. Neurosci. 15:625983. doi: 10.3389/fnhum.2021.625983, PMID: 34163337 PMC8215169

[ref8] Gao C. Sun J. (2022). Semi-supervised multi-source transfer learning for motor imagery recognition. Int. J. Pattern Recogn. 36:2250041. doi: 10.1142/S0218001422500410

[ref9] He H. Wu D. (2019). Transfer learning for brain-computer interfaces: a Euclidean space data alignment approach. IEEE Trans. Biomed. Eng. 67, 399–410. doi: 10.1109/TBME.2019.2913914, PMID: 31034407

[ref10] Hwang H.-J. Kwon K. Im C.-H. (2009). Neurofeedback-based motor imagery training for brain–computer interface (BCI). J. Neurosci. Methods 179, 150–156. doi: 10.1016/j.jneumeth.2009.01.015, PMID: 19428521

[ref11] Jeunet C. Jahanpour E. Lotte F. (2016). Why standard brain-computer interface (BCI) training protocols should be changed: an experimental study. J. Neural Eng. 13:036024. doi: 10.1088/1741-2560/13/3/036024, PMID: 27172246

[ref12] Ko W. Jeon E. Yoon J. S. Suk H. (2020). Semi-supervised generative and discriminative adversarial learning for motor imagery-based brain–computer interface. Sci. Rep. 12:4587. doi: 10.1038/s41598-022-08490-9, PMID: 35301366 PMC8931045

[ref13] Li X. Wang L. Miao S. Yue Z. Tang Z. Su L.. (2022). Sensorimotor rhythm-brain computer interface with audio-cue, motor observation and multisensory feedback for upper-limb stroke rehabilitation: a controlled study. Front. Neurosci. 16:808830. doi: 10.3389/fnins.2022.808830 35360158 PMC8962957

[ref14] Liu M. Zhou M. Zhang T. Xiong N. (2020). Semi-supervised learning quantization algorithm with deep features for motor imagery EEG recognition in smart healthcare application. Appl. Soft Comput. 89:106071. doi: 10.1016/j.asoc.2020.106071

[ref9001] Lotte F. Congedo M. Lécuyer A. Lamarche F. Arnaldi B. (2007). A review of classification algorithms for EEG-based brain–computer interfaces. J. Neural Eng. 4, R1–R13. doi: 10.1088/1741-2560/4/2/R01 17409472

[ref15] Lotte F. Guan C. (2011). Regularizing common spatial patterns to improve BCI designs: unified theory and new algorithms. IEEE Trans. Biomed. Eng. 58, 355–362. doi: 10.1109/TBME.2010.2082539 20889426

[ref16] Lotte F. Jeunet C. (2018). Defining and quantifying users’ mental imagery based BCI skills: a first step. J. Neural Eng. 15:046030. doi: 10.1088/1741-2552/aac577, PMID: 29769435

[ref17] Mane R. Chouhan T. Guan C. (2020). BCI for stroke rehabilitation: motor and beyond. J. Neural Eng. 17:041001. doi: 10.1088/1741-2552/aba162, PMID: 32613947

[ref18] Marcos-Martínez D. Martínez-Cagigal V. Santamaría-Vázquez E. Pérez-Velasco S. Hornero R. (2021). Neurofeedback training based on motor imagery strategies increases EEG complexity in elderly population. Entropy 23:1574. doi: 10.3390/e23121574, PMID: 34945880 PMC8700498

[ref19] Mladenović J. Frey J. Pramij S. Mattout J. Lotte F. (2022). Towards identifying optimal biased feedback for various user states and traits in motor imagery BCI. I IEEE Trans. Biomed. Eng. 69, 1101–1110. doi: 10.1109/TBME.2021.3113854, PMID: 34543189

[ref20] Pfurtscheller G. Flotzinger D. Kalcher J. (1993). Brain-computer interface—a new communication device for handicapped persons. J. Microcomput. Appl. 16, 293–299. doi: 10.1006/jmca.1993.1030

[ref21] Pfurtscheller G. Neuper C. Guger C. Harkam W. Ramoser H. Schlögl A.. (2000). Current trends in Graz brain-computer interface (BCI) research. IEEE Trans. Neural Sys. Rehabil. Eng. 8, 216–219. doi: 10.1109/86.847821, PMID: 10896192

[ref22] Qiu Z. Allison B. Z. Jin J. Zhang Y. Wang X. Li W.. (2017). Optimized motor imagery paradigm based on imagining Chinese characters writing movement. IEEE Trans. Neural. Syst. Rehabil. 25, 1009–1017. doi: 10.1109/TNSRE.2017.2655542, PMID: 28113345

[ref23] Ramoser H. Müller-Gerking J. Pfurtscheller G. (2000). Optimal spatial filtering of single trial EEG during imagined hand movement. IEEE Trans. Rehabil. Eng. 8, 441–446. doi: 10.1109/86.895946, PMID: 11204034

[ref24] She Q. Zhou Y. Gan H. Ma Y. Luo Z. (2019). Decoding EEG in motor imagery tasks with graph semi-supervised broad learning. Electronics 8:1273. doi: 10.3390/electronics8111273

[ref25] Škola F. Tinková S. Liarokapis F. (2019). Progressive training for motor imagery brain-computer interfaces using gamification and virtual reality embodiment. Front. Hum. Neurosci. 13:329. doi: 10.3389/fnhum.2019.00329, PMID: 31616269 PMC6775193

[ref26] Tharwat A. Schenck W. (2023). A survey on active learning: state-of-the-art, practical challenges and research directions. Mathematics 11:820. doi: 10.3390/math11040820

[ref27] Vasilyev A. N. Nuzhdin Y. O. Kaplan A. Y. (2021). Does real-time feedback affect sensorimotor EEG patterns in routine motor imagery practice? Brain Sci. 11:1234. doi: 10.3390/brainsci11091234, PMID: 34573253 PMC8469546

[ref28] Vidaurre C. Blankertz B. (2010). Towards a cure for BCI illiteracy. Brain Topogr. 23, 194–198. doi: 10.1007/s10548-009-0121-6, PMID: 19946737 PMC2874052

[ref29] Wan Z. Yang R. Huang M. Zeng N. Liu X. (2021). A review on transfer learning in EEG signal analysis. Neurocomputing 421, 1–14. doi: 10.1016/j.neucom.2020.09.017

[ref30] Wang Z. Zhou Y. Chen L. Gu B. Liu S. Xu M.. (2019). A bci based visual-haptic neurofeedback training improves cortical activations and classification performance during motor imagery. J. Neural Eng. 16:066012. doi: 10.1088/1741-2552/ab377d, PMID: 31365911

[ref31] Wolpaw J. R. Birbaumer N. McFarland D. J. Pfurtscheller G. Vaughan T. M. (2002). Brain-computer interfaces for communication and control. Clin. Neurophysiol. 113, 767–791. doi: 10.1016/S1388-2457(02)00057-3 12048038

[ref32] Wu D. Jiang X. Peng R. (2022). Transfer learning for motor imagery based brain-computer interfaces: a tutorial. Neural Netw. 153, 235–253. doi: 10.1016/j.neunet.2022.06.008, PMID: 35753202

[ref33] Xu Y. Huang X. Lan Q. (2021). Selective cross-subject transfer learning based on riemannian tangent space for motor imagery brain-computer interface. Front. Neurosci. 15:779231. doi: 10.3389/fnins.2021.779231, PMID: 34803600 PMC8595943

[ref34] Xu B. Li W. He X. Wei Z. Zhang D. Wu C.. (2020). Motor imagery based continuous teleoperation robot control with tactile feedback. Electronics 9:174. doi: 10.3390/electronics9010174

[ref35] Zanini P. Congedo M. Jutten C. Said S. Berthoumieu Y. (2018). Transfer learning: a riemannian geometry framework with applications to brain–computer interfaces. IEEE Trans. Biomed. Eng. 65, 1107–1116. doi: 10.1109/TBME.2017.2742541 28841546

[ref36] Zhang L. Li C. Zhang R. Sun Q. (2023). Online semi-supervised learning for motor imagery EEG classification. Comput. Biol. Med. 165:107405. doi: 10.1016/j.compbiomed.2023.107405, PMID: 37678137

[ref37] Zhang W. Song A. Zeng H. Xu B. Miao M. (2022). The effects of bilateral phase-dependent closed-loop vibration stimulation with motor imagery paradigm. IEEE Trans. Neural. Syst. Rehabil. 30, 2732–2742. doi: 10.1109/TNSRE.2022.3208312, PMID: 36129854

[ref38] Zhang W. Wu D. (2020). Manifold embedded knowledge transfer for brain-computer interfaces. IEEE Trans. Neural Syst. Rehabil. Eng. 28, 1117–1127. doi: 10.1109/TNSRE.2020.2985996, PMID: 32286993

[ref39] Zhao X. Zhao J. Cai W. Wu S. (2019). Transferring common spatial filters with semi-supervised learning for zero-training motor imagery brain-computer interface. IEEE Access 7, 58120–58130. doi: 10.1109/ACCESS.2019.2913154

